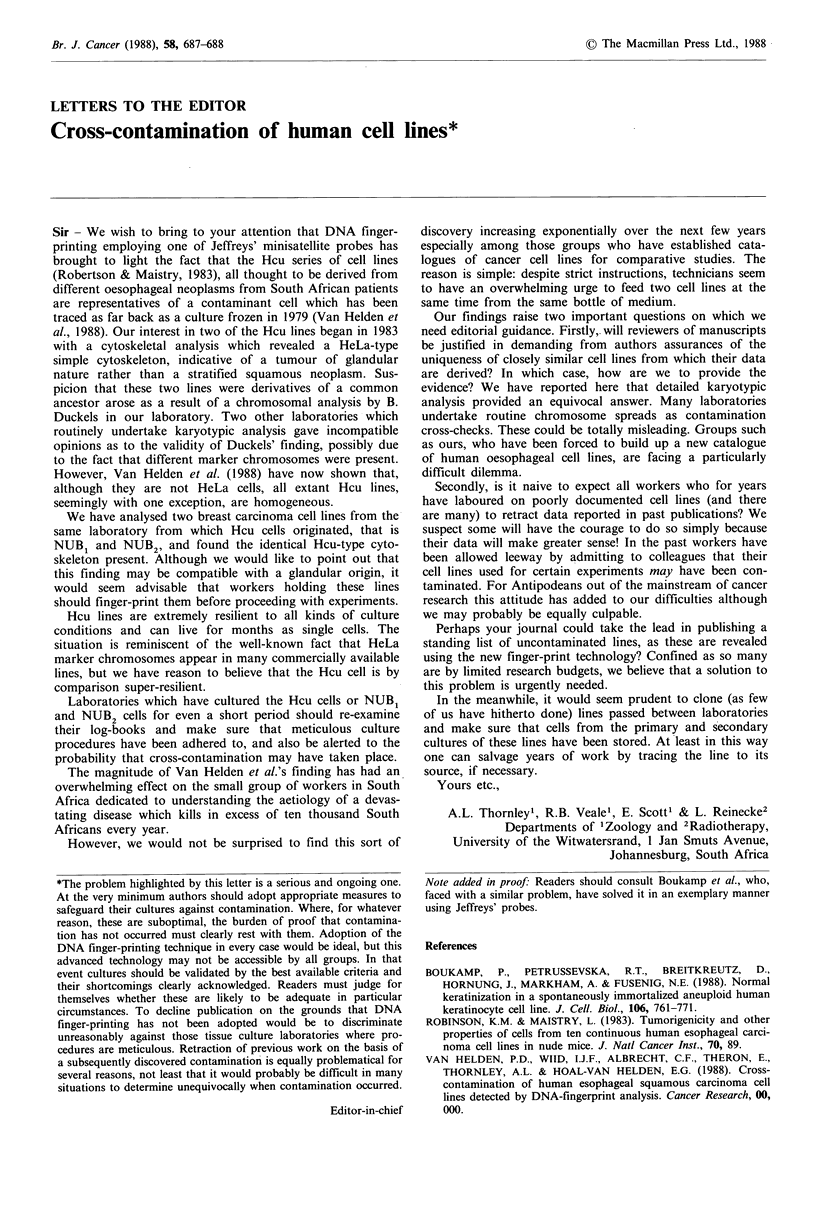# Cross-contamination of human cell lines.

**DOI:** 10.1038/bjc.1988.287

**Published:** 1988-11

**Authors:** A. L. Thornley, R. B. Veale, E. Scott, L. Reinecke


Br. J. Cancer (1988), 58, 687–688 © The Macmillan Press Ltd., 1988

### LETTERS TO THE EDITOR

### Cross-contamination of human cell lines*

**Sir** – We wish to bring to your attention that DNA finger-printing employing one of Jeffreys' minisatellite probes has brought to light the fact that the Hcu series of cell lines (Robertson & Maistry, 1983), all thought to be derived from different oesophageal neoplasms from South African patients are representatives of a contaminant cell which has been traced as far back as a culture frozen in 1979 (Van Helden *et al.*, 1988). Our interest in two of the Hcu lines began in 1983 with a cytoskeletal analysis which revealed a HeLa-type simple cytoskeleton, indicative of a tumour of glandular nature rather than a stratified squamous neoplasm. Suspicion that these two lines were derivatives of a common ancestor arose as a result of a chromosomal analysis by B. Duckels in our laboratory. Two other laboratories which routinely undertake karyotypic analysis gave incompatible opinions as to the validity of Duckels' finding, possibly due to the fact that different marker chromosomes were present. However, Van Helden *et al.* (1988) have now shown that, although they are not HeLa cells, all extant Hcu lines, seemingly with one exception, are homogeneous.

We have analysed two breast carcinoma cell lines from the same laboratory from which Hcu cells originated, that is $NUB_1$ and $NUB_2$, and found the identical Hcu-type cytoskeleton present. Although we would like to point out that this finding may be compatible with a glandular origin, it would seem advisable that workers holding these lines should finger-print them before proceeding with experiments.

Hcu lines are extremely resilient to all kinds of culture conditions and can live for months as single cells. The situation is reminiscent of the well-known fact that HeLa marker chromosomes appear in many commercially available lines, but we have reason to believe that the Hcu cell is by comparison super-resilient.

Laboratories which have cultured the Hcu cells or $NUB_1$ and $NUB_2$ cells for even a short period should re-examine their log-books and make sure that meticulous culture procedures have been adhered to, and also be alerted to the probability that cross-contamination may have taken place.

The magnitude of Van Helden *et al.*'s finding has had an overwhelming effect on the small group of workers in South Africa dedicated to understanding the aetiology of a devastating disease which kills in excess of ten thousand South Africans every year.

However, we would not be surprised to find this sort of discovery increasing exponentially over the next few years especially among those groups who have established catalogues of cancer cell lines for comparative studies. The reason is simple: despite strict instructions, technicians seem to have an overwhelming urge to feed two cell lines at the same time from the same bottle of medium.

Our findings raise two important questions on which we need editorial guidance. Firstly, will reviewers of manuscripts be justified in demanding from authors assurances of the uniqueness of closely similar cell lines from which their data are derived? In which case, how are we to provide the evidence? We have reported here that detailed karyotypic analysis provided an equivocal answer. Many laboratories undertake routine chromosome spreads as contamination cross-checks. These could be totally misleading. Groups such as ours, who have been forced to build up a new catalogue of human oesophageal cell lines, are facing a particularly difficult dilemma.

Secondly, is it naive to expect all workers who for years have laboured on poorly documented cell lines (and there are many) to retract data reported in past publications? We suspect some will have the courage to do so simply because their data will make greater sense! In the past workers have been allowed leeway by admitting to colleagues that their cell lines used for certain experiments *may* have been contaminated. For Antipodeans out of the mainstream of cancer research this attitude has added to our difficulties although we may probably be equally culpable.

Perhaps your journal could take the lead in publishing a standing list of uncontaminated lines, as these are revealed using the new finger-print technology? Confined as so many are by limited research budgets, we believe that a solution to this problem is urgently needed.

In the meanwhile, it would seem prudent to clone (as few of us have hitherto done) lines passed between laboratories and make sure that cells from the primary and secondary cultures of these lines have been stored. At least in this way one can salvage years of work by tracing the line to its source, if necessary.

Yours etc.,

A.L. Thornley[1], R.B. Veale[1], E. Scott[1] & L. Reinecke[2]

Departments of [1]Zoology and [2]Radiotherapy,

University of the Witwatersrand, 1 Jan Smuts Avenue,

Johannesburg, South Africa

*Note added in proof:* Readers should consult Boukamp *et al.*, who, faced with a similar problem, have solved it in an exemplary manner using Jeffreys' probes.

*The problem highlighted by this letter is a serious and ongoing one. At the very minimum authors should adopt appropriate measures to safeguard their cultures against contamination. Where, for whatever reason, these are suboptimal, the burden of proof that contamination has not occurred must clearly rest with them. Adoption of the DNA finger-printing technique in every case would be ideal, but this advanced technology may not be accessible by all groups. In that event cultures should be validated by the best available criteria and their shortcomings clearly acknowledged. Readers must judge for themselves whether these are likely to be adequate in particular circumstances. To decline publication on the grounds that DNA finger-printing has not been adopted would be to discriminate unreasonably against those tissue culture laboratories where procedures are meticulous. Retraction of previous work on the basis of a subsequently discovered contamination is equally problematical for several reasons, not least that it would probably be difficult in many situations to determine unequivocally when contamination occurred.

Editor-in-chief

### References

BOUKAMP, P., PETRUSSEVSKA, R.T., BREITKREUTZ, D., HORNUNG, J., MARKHAM, A. & FUSENIG, N.E. (1988). Normal keratinization in a spontaneously immortalized aneuploid human keratinocyte cell line. *J. Cell. Biol.*, **106**, 761–771.

ROBINSON, K.M. & MAISTRY, L. (1983). Tumorigenicity and other properties of cells from ten continuous human esophageal carcinoma cell lines in nude mice. *J. Natl Cancer Inst.*, **70**, 89.

VAN HELDEN, P.D., WIID, I.J.F., ALBRECHT, C.F., THERON, E., THORNLEY, A.L. & HOAL-VAN HELDEN, E.G. (1988). Cross-contamination of human esophageal squamous carcinoma cell lines detected by DNA-fingerprint analysis. *Cancer Research*, **00**, 000.